# Petrologic Characteristics of the Lunar Surface

**DOI:** 10.1038/srep17075

**Published:** 2015-11-27

**Authors:** Xianmin Wang, Witold Pedrycz

**Affiliations:** 1 Hubei Subsurface Multi-scale Imaging Key Laboratory, Institute of Geophysics and Geomatics, China University of Geosciences, Wuhan, 430074, China; 2 Department of Electrical and Computer Engineering, University of Alberta, Edmonton, T6R 2V4, AB, Canada; 3 Department of Electrical and Computer Engineering, Faculty of Engineering, King Abdulaziz University, Jeddah, 21589, Saudi Arabia; 4 Systems Research Institute, Polish Academy of Sciences, Warsaw, Poland

## Abstract

Petrologic analysis of the lunar surface is critical for determining lunar formation and evolution. Here, we report the first global petrologic map that includes the five most important lunar lithological units: the Ferroan Anorthositic (FAN) Unit, the Magnesian Suite (MS) Unit, the Alkali Suite (AS) Unit, the KREEP Basalt (KB) Unit and the Mare Basalt (MB) Unit. Based on the petrologic map and focusing on four long-debated and important issues related to lunar formation and evolution, we draw the following conclusions from the new insights into the global distribution of the five petrologic units: (1) there may be no petrogenetic relationship between MS rocks and KB; (2) there may be no petrogenetic link between MS and AS rocks; (3) the exposure of the KREEP component on the lunar surface is likely not a result of MB volcanism but is instead mainly associated with the combined action of plutonic intrusion, KREEP volcanism and celestial collision; (4) the impact size of the South Pole-Aitken basin is constrained, i.e., the basin has been excavated through the whole crust to exhume a vast majority of lower-crustal material and a very limited mantle components to the lunar surface.

Lunar surface materials were thought to originate from the lunar crust and mantle and brought to the surface by evolutionary processes such as Lunar Magma Ocean (LMO) crystallization, celestial impact and volcanism^1^,^2^. Therefore, characterizing the petrologic features of the lunar surface is a key to understanding lunar formation and evolution and to determining the function of lunar volcanism and impact excavation, but also is a core to thoroughly understanding lunar chemical heterogeneity. Furthermore, the mare basalts (MBs) and KREEP basalts (KBs) on the lunar surface also provide important mineral resources^2^.

At present, research on the petrologic analysis of the lunar surface primarily comprises four aspects. First, some researchers have focused on using a two- or three-dimensional element space to analyze the characteristics of various lunar rocks 3,4,5,6,7. For example, a few studies have suggested that feldspathic rocks, MBs and KBs appear as a triangular structure in the two-dimensional Fe-Th or Fe-(Th/Ti) space^3^,^7^,^8^. Berezhnoy *et al.*^5^ exploited a pixel distance map of the three-dimensional Mg-Al-Fe space to identify chemical abnormalities on the lunar surface. Second, some studies have divided the lunar surface into specific regions based on the chemical and physical features. Considering the FeO and Th abundances, Jolliff *et al.*^8^ expressed the lunar surface in terms of three geological provinces: the Procellarum KREEP Terrane (PKT), the Feldspathic Highlands Terrane (FHT) and the South Pole-Aitken Terrane (SPAT). Joshua *et al.*^9^ segmented the lunar surface into four regions: the Nearside Radar Dark Terrane, the Orientale Impact Basin Terrane, the Feldspathic Highlands Terrane, and the South Pole-Aitken (SPA) basin Terrane. Third, some works have employed remote sensing data to identify the distribution areas of three or four types of rocks (e.g., plagioclases, Mg-rich rocks, MBs and KBs) on the lunar surface^10^,^11^,^12^. Li *et al.*^10^ and Du *et al.*^11^ adopted three elements—Fe, Mg and Th—to partition the lunar surface into four rock units. Wöhler *et al.*^12^ employed the Mg-Fe space and Mg-Al space to determine the distribution of three lithology types: MB, Mg-rich rock and ferroan anorthosite (FAN). Fourth, many research studies have investigated their analysis to a single rock type, e.g., low-Ca pyroxene or plagioclase, or a local area on the moon, e.g., Mare Moscoviense or the Oriental Basin^13^,^14^,^15^,^16^,^17^,^18^,^19^.

On the lunar surface, there are four primary crustal rock types (FAN suite, magnesian suite (MS), alkali suite (AS) and KBs) and one primary mantle rock type (MBs)^2^. Volcanic glasses also represent the mantle components^2^ which is not included in this study due to the lack of their specific Th contents in “New Views of the Moon”^2^. Polymict breccias and lunar soils are mixing materials from fragments of the primary rock types^2^,^20^. Therefore, according to the petrologic characteristics and chemical composition, we propose to divide the lunar surface into five geological units: the FAN Unit, the MS Unit, the AS Unit, the KB Unit and the MB Unit. Each locale on the moon is attributed to the corresponding geological unit based on its dominant petrologic feature. This paper makes five major contributions: (1) the first global petrologic map, which includes five geological units and reflects, at the macro level, the distribution of the most important rock types on the moon. (2) The abundances of six oxides or elements (TiO_2_, Al_2_O_3_, FeO, MgO, CaO and Th), as the signature of lunar primary rocks, are employed to generate the petrologic map, which makes the map more precise. (3) By superimposing the petrologic map on LRO LOLA elevation data, the global distribution characteristics of five geological units on the lunar surface are discussed. (4) Three long-debated and important issues are analyzed: Is there a petrogenetic link between MS rocks and KB? Is there a petrogenetic relationship between MS and AS rocks? What is the evolutionary process that brought KREEP materials to the lunar surface? (5) The petrologic features and impact size of the South Pole-Aitken (SPA) basin, a particular area on the moon, are constrained from the new insights into the distribution of five geological units.

## Results

**Figure 1:**
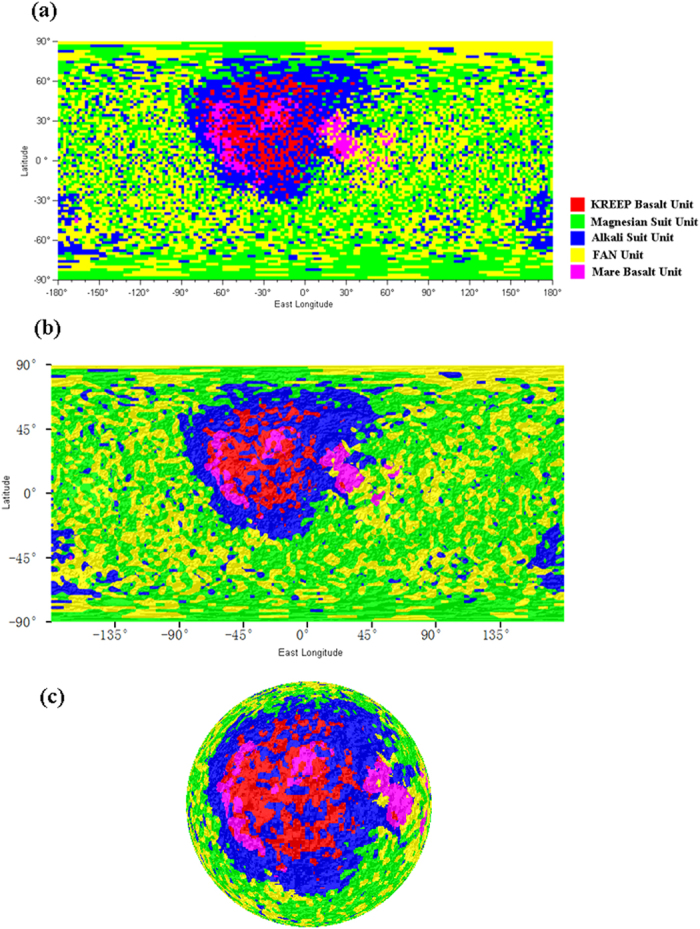
Petrologic maps of the lunar surface at a macro level. All the maps are cylindrically projected at a resolution of 2° × 2°. (**a**) Petrologic distribution on the lunar surface. (**b**) Smoothed petrologic units superimposed on the shaded relief. The shaded lunar surface relief is produced based on LRO LOLA DEM data^25^. (**c**) Petrologic features on the lunar nearside with the shaded lunar surface relief.

Two data sets are adopted to determine the petrologic distribution on the lunar surface: the lunar rock samples from “New Views of the Moon”^2^ and the abundance data of major oxides and elements of the whole lunar surface^4^,^21^, which were extracted from Lunar Prospector (LP) Gamma Ray and Neutron Spectrometer (GRNS) data and released in 2012. Detailed descriptions of our methods and procedures are provided in the Methods section. For convenience, the procedure is briefly stated here. First, according to the chemical component characteristics of the five geological units, six key oxides and elements (TiO_2_, Al_2_O_3_, FeO, MgO, CaO and Th) are selected as the lithology signature. Second, based on the selected six oxides and elements, six petrologic indexes (Table 1) are defined to better distinguish the petrologic features of various lithologies. Third, using the six petrologic indexes of lunar rock samples as the training data, a decision tree algorithm and boosting technique^22^,^23^,^24^ are adopted to construct the lithology identification model. Fourth, according to the lithology identification model and a knowledge-based mechanism^22^, the six petrologic indexes of the whole lunar surface, which were derived from LP data, are input to identify the five petrologic units on the lunar surface. The petrologic maps of the lunar surface are shown in Fig. 1. To verify the accuracy of the petrologic map (Fig. 1(a)) and the lithology identification model, the lunar rock samples^2^ are used as validation data by running 10-fold cross-validations 10 times. The obtained validation accuracies are 87.4%, 89.2%, 86.4%, 90.1%, 89.2%, 88.4%, 89.2%, 87.3%, 90.2%, and 89.3%. To improve the classification result, the median filtering method with a window size of 7*7 is employed to smooth the classification map (Fig. 1(a)) during the post-processing of the classification; the smoothed petrologic map is shown in Fig. 1(b).

**Figure 2:**
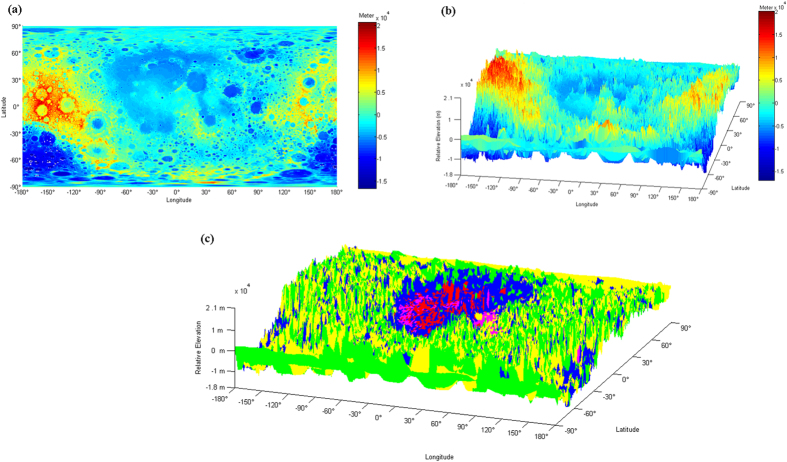
Three-dimensional map of petrologic units based on LRO LOLA DEM data. (**a**) Two-dimensional map of lunar elevations (relative to a radius of 1737.4 km) from the LRO LOLA^25^ binned at a resolution of 0.5° × 0.5°. (**b**) Three-dimensional map of lunar relative topography from the LRO LOLA^25^ binned into 0.5° pixels of equal area. (**c**) Three-dimensional distribution of five petrologic units (the FAN unit, the MS Unit, the AS Unit, the KB Unit and the MB Unit) superimposed on LRO LOLA DEM data^25^ at a resolution of 2°.

The petrologic map is superimposed on the LRO LOLA DEM (Digital Elevation Model) data^25^ binned at a resolution of 0.5° × 05° (Fig. 2), with minimum and maximum relative elevations of −17.003 km and 20.061 km, respectively. Note that the map height values are relative to a radius of 1737.4 km, i.e., the LRO LOLA DEM data show a relative topography^25^. The elevation ranges of various petrologic units (Table 2) indicate that the KB Unit and MB Unit are distributed at lower elevations. Although the generated petrologic map is binned at a resolution of 0.5° × 05°, its actual resolution is still 2° × 2° because the highest resolution of the elemental abundance data of the whole lunar surface^4^,^21^, which were derived from LP, is 2° × 2°. Note our work is limited by the detection precision and spatial resolution of the LP instruments and also limited by the sites where the lunar rock samples were returned. Furthermore, in the future work, the inclusion of the volcanic glass group might further improve the accuracy of the petrologic map.

## Discussion

In terms of the petrologic map (Fig. 1), we present a discussion and analysis of the following four aspects: (1) the distribution characteristics of three petrologic units, i.e., the FAN Unit, the MS Unit and the AS Unit; the petrologic relationship between Mg-rich rocks and KB; and the petrologic connection between Mg-rich rocks and AS; (2) the exposure area of the KB on the lunar surface and the excavation and exposure reason of the KREEP component; (3) the exposed region of MB; and (4) the constraints on the rock types and impact size of the SPA basin.

As shown in the petrologic map (Fig. 1), the FAN and MS Units are the most widely distributed over the lunar surface. They show a global distribution outside the central regions of PKT and SPAT. The mixed distribution of the FAN and MS units indicate that MS magmatism may have been a global phenomenon, causing global intrusion into the early lunar FAN crust during the evolution of the LMO^1^. Some studies^2^,^26^ have challenged our above point regarding a global distribution of the MS Unit (or the spatial relevance of the FAN and MS Units), e.g., by feldspathic lunar meteorites^2^, which primarily appear ferroan-anorthositic. However, the lunar meteorite samples are too few to represent the extensive highland region. Furthermore, non-mare lunar samples appear abundant with both the FAN and MS^2^. Some recent studies^15^,^17^ have validated our point by suggesting that MS magmatism may have globally occurred and that the purest anorthosite rock is widely distributed outside the central areas of the PKT and SPAT. Regarding the formation connection between Mg-rich rocks and KB, some studies^2^,^8^,^27^ have proposed that there is a petrogenetic relationship between the MS and KB; however, other studies^15^,^28^ have contradicted the above viewpoint, instead indicating that KREEP is not necessary for MS formation. In our study (Fig. 1), we discovered that the spatial distributions of the MS and KB are quite separate. The MS is primarily distributed outside the PKT area, but KREEP materials are confined within PKT. Hence, we report no petrogenetic link between the MS and KB. Based on the petrologic map (Fig. 1), we also draw the conclusion that there may be no petrogenetic connection between the MS and AS according to the divergence of their distribution features. The AS Unit is primarily enriched around the periphery of the PKT and in the center of SPAT, whereas the MS is evenly and globally distributed outside the central regions of the PKT and SPAT. Some study^26^ has also described an alternative model in which the MS and AS are genetically separate. Thus, the long-standing question^2^ regarding why the materials of the MS and AS are difficult to obtain together from crystalline breccias can be answered: they are genetically disconnected and separately distributed, as mentioned above.

The source region of KREEP materials is a transitional zone between the feldspathic crust and the mafic mantle^1^,^2^. Recent studies^6^,^8^,^10^,^29^ identified a region on the lunar surface as being the general exposure area of KREEP materials (or the Procellarum KREEP Terrane), which mostly contain Th contents exceeding a certain threshold. Our study incorporated six key elements (TiO_2_, Al_2_O_3_, FeO, MgO, CaO and Th), including Th, to identify the distribution region of KREEP materials on the moon and, hopefully, thereby increasing the accuracy of the results. As shown by the three-dimensional map of the petrologic units (Fig. 2), the KB Unit is primarily concentrated in the highland or mountain region encompassing the Imbrium Basin. Thus, the following question arises: Given that KREEP materials are located between the crust and mantle, how were they brought to and exposed on the lunar surface? Many studies have focused on how KREEP materials were excavated to the lunar surface. Three main mechanisms of KREEP component denudation have been proposed^30^: (1) volcanism and basaltic eruption spraying the KREEP materials onto the lunar surface^26^,^31^,^32^; (2) impact excavation of celestial bodies, which excavated into the KB magma^29^,^30^,^33^,^34^; and (3) invasion of KREEP-rich lavas into the crust and exposure of KREEP materials on the lunar surface by large meteorite impacts^35^,^36^. In our research, the unique distribution characteristics of the KB Unit led to the following deduction. If KREEP exposure was triggered by MB volcanism, the erupted MB magma should have flowed into the basins along with the KREEPy materials. Thus, the KB Unit should have been distributed relatively uniformly within the basin areas. However, the KB Unit is mainly enriched in the highland area, whereas closer to the basin center, fewer KREEP materials are exposed. Thus, no link between mare volcanism and KREEP denudation is believed to exist. We hold the similar point with some studie^29^ that after a long history of plutonic intrusion into the lower crust, the KREEP materials were excavated and ejected to the surrounding highland and mountain by KREEP volcanism and the impact excavation of celestial bodies.

As shown by the petrologic map, the MB Unit is primarily located in basins, such as Oceanus Procellarum, Imbrium, Serenitatis, Tranquillitatis, Crisium, Fecunditatis, Humourum and Vaporum. Thus, MB is not present on the top of all the basins and is actually absent on the surface of many basins. The viewpoint is supported by a few studies^26^,^37^, which proposed that the thickness of the lunar crust and the existence of basins cannot alone account for MB volcanism and eruptions. Both the atomic mass and neutron number density^4^ across the lunar surface, as determined by the Lunar Prospector, verified that the above-listed basins possess different chemical compositions compared with many of other basins. Some studies have indicated that the surfaces of many basins on the moon exhibit nonmare materials. Hagerty *et al.*^38^ suggested that many basins are covered with nonmare mafic materials in SPAT. Mercer *et al.*^39^ reported that the surfaces of some basins, e.g., the Orientale Basin and Australe Basin, have a composition similar to that of the Northwest Africa (NWA) 2996 meteorite, which is rich in noritic and troctolitic anorthosite and magnesian pyroxenes but short of basalt. Bhattacharya *et al.*^13^ proposed that in the central region of the Mare Moscoviense, except for the ancient mare unit (Im), which appears compositionally gabbroic, all other mare units primarily present a composition dominated by noritic to anorthositic norite. Moreover, most current research studies^10^,^19^,^40^,^41^,^42^ mainly have employed the FeO content (or Fe^2+^ absorption features in olivines and pyroxenes near 1000 nm and 2000 nm) to determine (or analyze) MB and have identified the area with high FeO abundance or with some absorption feature as the exposure region of MB. However, from the chemical compositions of lunar rocks^2^,^43^, some quartz monzodiorites (monzogabbro) and some Mg-gabbronorites were also shown to have high FeO concentrations similar to those of MB. Hallis *et al.*^44^ suggested that MB cannot be identified on the basis of single source mineralogy. Thus, the determination of MBs according to the FeO content or Fe^2+^ absorption features alone may not be accurate. In our study, six important chemical compositions, serving as key proxies of various lithologies, are adopted to confirm the identity of the MB Unit; therefore, our result is likely to be more precise. However, it should be noted that the precision of our results is subject to the detector accuracy and spatial resolution of the LP data, and FeO abundance values derived from hyperspectral data may be more accurate in determining the distribution area of MB. Because the hyperspectral data, e.g., Interference Imaging Spectrometer (IIM), Moon Mineralogy Mapper (M^3^) and Clementine data, do not cover the complete lunar surface, the synthesis of Fe^2+^ absorption features derived from various hyperspectral images will be pursued in future work. Furthermore, Bugiolacchi *et al.*^45^ indicated that the comprehensive analysis of multiple spectral characteristics representing mineral components provides a good method to infer the diversity of MBs. Moreover, the identification of the MB Unit in our study is also limited by the sites where the lunar rock samples were collected.

**Figure 3:**
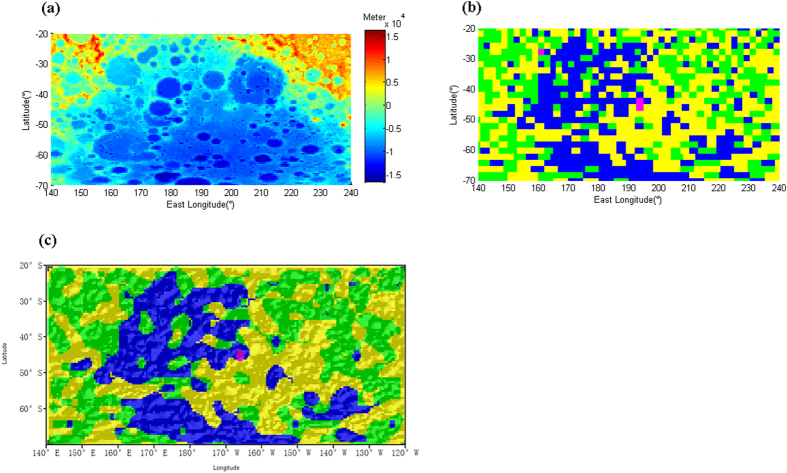
Petrologic maps of the SPA basin floor in a cylindrical projection with a resolution of 2° × 2°. (**a**) Topography of the SPA basin from LRO LOLA DEM data^25^ binned into a 0.5° × 0.5° resolution. (**b**) Petrologic units on the SPA basin floor. (**c**) Smoothed petrologic distribution on the SPA basin floor by the median filtering method with a window size of 7*7. The shaded relief was generated from the topographic data of LRO LOLA^25^.

The rock types and impact size of the South Pole-Aitken basin have historically been hotly debated issues. Some studies^46^,^47^,^48^have indicated that the upper-mantle basaltic materials were excavated, mixed with the noritic lower-crust component and widely or partially distributed on the SPA basin floor. Some researcher^49^ has proposed that the impact cratering in the SPA basin might not have excavated into the mantle; thus, the exposed materials constituting the floor of the basin should have dominantly originated from the lower crust. Some works^2^,^38^,^50^ have suggested that relatively little MB is present on the basin floor. In our study, the petrologic characteristics of the SPA basin (Fig. 3) indicate that the basin floor is dominated by three petrologic units: the AS Unit, the FAN unit and the MS Unit. Additionally, the majority of the central region is dominated by AS. To answer the question of whether the SPA basin has been excavated to the mantle, i.e., the impact size of the SPA basin, we need to first focus on the moderate thorium abundance and AS in the SPA basin^4^,^38^. Trace-element enrichment is a signature of AS^2^. Therefore, our work sheds light on the Th enhancement present in the basin floor^4^,^38^, i.e., AS is responsible for the elevated Th abundance. We propose that the AS might have originated from the more mafic lower crust based on three reasons. The first is that alkali norite and gabbronorite are mafic and that the lower-crustal materials are more mafic than the upper materials^2^. The second is that the SPA basin floor is thought to be mainly composed of lower-crustal materials^2^ and, as mentioned above, the AS dominates the majority of the center of the SPA basin floor. The third reason is that the signature of Th enrichment in the AS^2^,^43^ indicates a lower crust origin. Compared with other lunar rock types, KB and AS show typical Th concentrations^2^,^43^, and KREEP materials are sandwiched in the area between the crust and mantle^1^,^2^. Therefore, AS, which possesses a Th enrichment similar to that of KREEP materials, was very likely produced in the lower crust. Moreover, a higher quantity of heat-producing elements is located in the crust than in the mantle^8^. Thus, we deduce that the source region of alkali norite and gabbronorite might lie in the lower crust. In addition to AS, to determine MS in the SPA basin, some studies^5^,^14^,^28^ have proposed that there are Mg-rich lithologies in the SPA basin. Regarding the MB Unit, as Hagerty *et al.*^38^ and Elkin-Tanton *et al.*^50^ suggested, we found a small amount of MB present on the SPA basin floor that was derived from the lunar mantle. Therefore, the SPA basin is believed to have excavated through the whole crust^47^. A vast majority of lower crustal materials and a very limited mantle component are exposed on the floor.

## Methods

**Figure 4:**
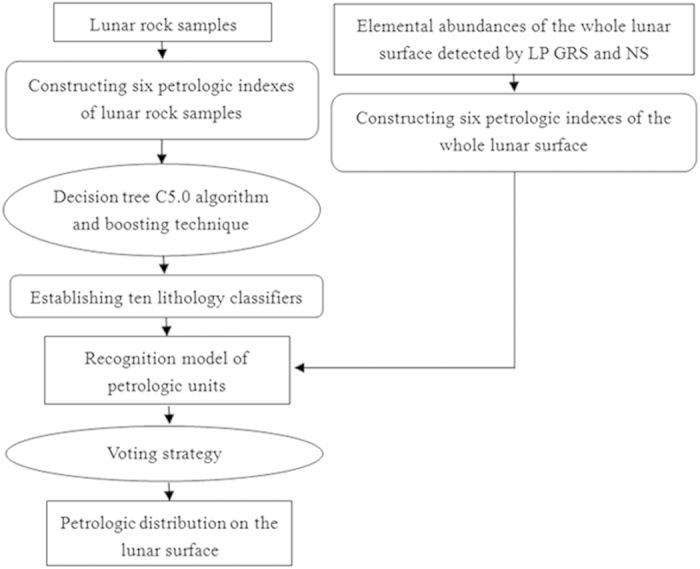
Flow diagram of the petrologic analysis of the lunar surface.

The petrologic characteristics of the lunar surface are determined via three steps: (1) the choice of oxides and elements, (2) the establishment of petrologic indexes, and (3) the determination of the petrologic distribution on the lunar surface. Our method and procedure are shown in Fig. 4.

In the first step, six key oxides and elements (TiO_2_, Al_2_O_3_, FeO, MgO, CaO and Th) are chosen as the lithology signature. The lithology-signature selection is based on four factors: (a) The abundances of nine important elements are obtained from LP GRS (Gamma Ray Spectrometer) and Neutron Spectrometer (NS) data, including TiO_2_, Al_2_O_3_, FeO, MgO, CaO, Th, SiO_2_, K and U^4^,^21^. (b) The U content in the above LP dataset is linked to that of Th as follows^4^,^21^: U wt.% = 0.27 × Th wt.%, i.e., the U and Th contents possess a strong linear correlation. Thorium abundance is a very important tool for lunar lithology identification; thus, the element U is eliminated, and Th is retained. (c) Accurate concentrations of SiO_2_ and K are unavailable for a certain number of lunar rock samples^2^,^43^. Therefore, SiO_2_ and K are excluded, and the remaining six oxides and elements (TiO_2_, Al_2_O_3_, FeO, MgO, CaO and Th) are chosen. (d) The chemical component characteristics of various petrologic units are also included to confirm that the above six elements can act as lithology signatures and proxies. The FAN suite has high aluminum and calcium contents and quite low incompatible-element abundances^2^,^51^. The MS is characterized by its high Mg/Fe ratio compared with the anorthite concentration^2^. The AS is distinguished by its alkali-element enrichment and particular trace-element pattern^2^. The KB possesses a unique trace-element enrichment and appears somewhat aluminous^2^. The Th concentration is an important signature for recognizing KB^8^. Finally, MB can be identified by its low incompatible-element abundance, especially its Th content, and its high FeO concentration^2^. FeO and TiO_2_ abundances are important compositional markers in identifying MB^2^. Therefore, according to the petrologic characteristics of various lunar lithologies, six key oxides and elements (TiO_2_, Al_2_O_3_, FeO, MgO, CaO and Th) are selected as the lithology signature for the petrologic analysis.

In the second step, six petrologic indexes are established, as shown in Table 1. The development of the petrologic indexes is motivated by the following three reasons: (a) Relative to the absolute abundances, the content ratios of oxides or elements can better indicate the petrologic features and mineral and compositional structures of lunar lithologies and can eliminate the resolution difference between the LP measurements and the lunar rock samples. (b) In the elemental abundance data detected by LP GRS and NS^4^,^21^, only one sample has zero CaO content; thus, the abundance of CaO can be used as the divisor. We define a Euclidean distance of compositional abundances to weigh the abundance difference between two samples. For the sample with zero CaO content, the values of the various petrologic indexes, except Mg/(Mg + Fe), are chosen as the values of one of its four adjacent samples, which possess the minimum abundance difference from the sample with zero CaO content. (c) We use a support vector machine (SVM) to judge the contributions of various compositions to lithology recognition, and the importance values of various components are shown in Table 3. CaO is relatively less important for petrologic unit determination. Considering the above points, six petrologic indexes are developed.

In the last step, two data sets are used: lunar rock samples and their chemical compositions^2^,^43^ as well as the elemental abundances of the whole lunar surface detected by LP GRS and NS^4^,^21^. First, six petrologic indexes of lunar rock samples are used as training samples, and the decision tree C5.0 algorithm^22^ is employed to construct the lithology classifier. The decision tree includes three types of nodes^22^: a root node, internal nodes and leaf nodes. Each internal node indicates a certain petrologic index (PI) and its related threshold *T*. Two branches—the PI value > *T* and PI value ≤ *T*—are extended from each internal node. The information gain ratio^22^ is employed to determine the priorities of various attributes (petrologic indexes). Each leaf node represents a class, i.e., a petrologic unit. Hence, a classification rule can be deduced by traversing from the root node to each leaf node^22^. Here, we present a classification rule as an example: “If Mg/(Mg + Fe) ≤ 0.66, Th/CaO ≤ 0.383 and FeO/CaO > 1.297, then this lunar unit is attributed to the MB Unit.” Thus, all the classification rules constitute the lithology classifier. Second, the six petrologic indexes of the whole lunar surface, which are established in terms of the elementary abundance detected by LP GRS and NS, are input into the constructed lithology classifier to identify the petrologic units on the lunar surface. For each locale on the lunar surface, its six petrologic index values are input into each decision tree to search for the corresponding unique classification rule according to a knowledge-based mechanism. The conclusion of the unique classification rule indicates the petrologic unit that this locale is a member of. Moreover, during the above two procedures, the boosting technique and voting mechanism^22^,^23^,^24^ are adopted to obtain a more accurate petrologic distribution. We set the number of trials to be 10, in which 10 classification trees are established with estimated accuracies of 97.3%, 88.92%, 94.28%, 98.09%, 83.5%, 93.12%, 95.66%, 88.86%, 85.23%, and 85.46%. Then, the final classification result of the petrologic units of the lunar surface is a synthetic one that takes the results of the above 10 decision trees into consideration using a voting strategy.

Our method possesses two advantages: First, the six key lithology signatures (TiO_2_, Al_2_O_3_, FeO, MgO, CaO and Th) are included to represent petrologic characteristics. Second, a data-mining method is employed to determine the petrologic distribution. Based on these two advantages, our result is likely relatively accurate. However, it should be noted that our work is limited by the detection precision and data resolution of the LP data and also limited by the sites where the lunar rock samples were returned.

## Additional Information

**How to cite this article**: Wang, X. and Pedrycz, W. Petrologic Characteristics of the Lunar Surface. *Sci. Rep.* **5**, 17075; doi: 10.1038/srep17075 (2015).

## Figures and Tables

**Table 1:** Six petrologic indexes established to identify various petrologic units.

Six Petrologic Indexes	Unit
TiO_2_/CaO	wt.%/wt.%
Al_2_O_3_/CaO	wt.%/wt.%
FeO/CaO	wt.%/wt.%
MgO/CaO	wt.%/wt.%
Mg/(Mg + Fe)	wt.%/wt.%
Th/CaO	ppm/wt.%

**Table 2:** Topographic characteristics of five petrologic units. The elevation values are relative to a radius of 1737.4 km.

Region of Relative Elevation	FAN Unit	MS Unit	AS Unit	KB Unit	MB Unit
Minimum Relative Elevation (km)	−17.003	−15.562	−16.752	−9.482	−10.326
Maximum Relative Elevation (km)	20.061	18.058	18.822	4.606	1.663

**Table 3:** Importance values of various key oxides and elements.

Oxide or Element	Importance Value
FeO	0.404
Th	0.299
Al_2_O_3_	0.101
Mg/(Mg+Fe)	0.101
TiO_2_	0.077
CaO	0.018
MgO	0
